# Pertussis Toxin: A Key Component in Pertussis Vaccines?

**DOI:** 10.3390/toxins11100557

**Published:** 2019-09-21

**Authors:** Kelsey A. Gregg, Tod J. Merkel

**Affiliations:** Division of Bacterial, Parasitic and Allergenic Products, Center for Biologics Evaluation and Research, U.S. Food and Drug Administration, 10903 New Hampshire Ave, Silver Spring, MD 20993, USA; Kelsey.Gregg@fda.hhs.gov

**Keywords:** *Bordetella pertussis*, bacterial infection, immunization, pertussis toxin, whooping cough, pertussis, pertussis vaccine

## Abstract

*B. pertussis* is a human-specific pathogen and the causative agent of whooping cough. The ongoing resurgence in pertussis incidence in high income countries is likely due to faster waning of immunity and increased asymptomatic colonization in individuals vaccinated with acellular pertussis (aP) vaccine relative whole-cell pertussis (wP)-vaccinated individuals. This has renewed interest in developing more effective vaccines and treatments and, in support of these efforts, defining pertussis vaccine correlates of protection and the role of vaccine antigens and toxins in disease. Pertussis and its toxins have been investigated by scientists for over a century, yet we still do not have a clear understanding of how pertussis toxin (PT) contributes to disease symptomology or how anti-PT immune responses confer protection. This review covers PT’s role in disease and evidence for its protective role in vaccines. Clinical data suggest that PT is a defining and essential toxin for *B. pertussis* pathogenesis and, when formulated into a vaccine, can prevent disease. Additional studies are required to further elucidate the role of PT in disease and vaccine-mediated protection, to inform the development of more effective treatments and vaccines.

## 1. Introduction

*Bordetella pertussis* is a Gram-negative pathogen that causes pertussis, or whooping cough, a highly contagious disease spread by respiratory droplets [1]. It is a strict human pathogen with no other known reservoir. The disease has an initial catarrhal-phase of one to two weeks followed by four or more weeks of paroxysmal coughing. The severe coughing bouts are often followed by an inspiratory whoop, for which the disease is named [2]. The disease is generally afebrile and causes lymphocytosis. Other more severe symptoms can include post-tussive vomiting, apnea, cyanosis, seizures, encephalopathy, and weight loss [2]. The disease is milder in older children and adults and most severe in infants, with half of all deaths in the US occurring in infants below two months of age [3]. The bacterium that causes pertussis was first isolated by Bordet and Gengou in 1906 [4]. By 1914, multiple whole-cell pertussis (wP) vaccines were in use with variable efficacy [5,6]. Different *B. pertussis* strains and culture conditions reduced the expression of virulence factors, contributing to the variable efficacy of these early pertussis vaccines. The discovery of different antigenic growth phases and phase-locked mutants of *B. pertussis* [7] lead to more standardized growth conditions for wP vaccine production [8]. Widespread use of standardized wP vaccines, that also include diphtheria (D) and tetanus (T) toxoids, began in 1944 following the recommendations of the American Academy of Pediatrics [5,6]. Doctors and parents around the world became more concerned with the reactogenicity of wP vaccines as rates of disease fell, prompting the development of less-reactive acellular pertussis (aP) vaccines in the 1980s (please see Ligon [9] and Pittman [6] for more in-depth histories of this topic). Currently, almost all licensed wP and aP vaccines are combination vaccines that include D and T [10,11]. Many *B. pertussis* virulence factors were identified as research on pertussis progressed in the 20^th^ and beginning of the 21^st^ century, however, despite over a century of scientific inquiry, we still do not have a clear understanding of which antigens in the wP vaccine confer protection or the mechanisms underlying that protection. This review will cover pertussis toxin (PT) and its role in aP vaccines. With a better understanding of how this key toxin contributes to disease, better correlates of protection and a more effective vaccine may be developed. This information is increasingly important because of the increasing incidence of pertussis in high income countries despite high vaccination rates with current vaccines. 

## 2. Pertussis Toxin

*B. pertussis* establishes itself on ciliated cells in the conducting airways of the respiratory tract [12,13] and is not known to disseminate systemically in humans, although Scanlon et al. observed *B. pertussis* dissemination in an immunocompetent neonatal mouse model [14]. Non-systemic *B. pertussis* infections can have profound systemic effects—many of which are attributed to PT. PT is an ADP-ribosyltransferase that ribosylates inhibitory Gα_i_ subunits of G protein-coupled receptors (GPCRs) [15], that are involved in cell-signaling pathways throughout the body. This ribosylation permanently inactivates Gα_i_ subunits, removing the negative regulatory function of these inhibitory GPCRs causing an increase in the second messenger cyclic adenosine monophosphate (cAMP) and, for some GPCRs, also altering potassium and calcium channels [15]. Downstream effects of PT include leukocytosis [16], impaired macrophage function [17], altered leukocyte trafficking [18], hyperinsulinemia [19], and sensitivity to multiple agents, including histamine [20,21,22], bradykinin [23], and serotonin [22]. Because of these pleiotropic effects, this toxin originally went by a variety of different names, including lymphocyte-leukocyte-promoting factor hemagglutinin, histamine-sensitizing factor, islet-activating protein, and pertussigen before Dr. Margaret Pittman proposed the name PT. She hypothesized that pertussis was primarily a PT-mediated disease and immunity to the toxin conferred immunity to disease [24]. Continued research has revealed a more complex picture of pertussis pathogenesis and virulence factors [25,26] and adhesins such as fimbrial hemagglutinin (FHA), and fimbrial proteins 2/3 (Fim 2/3), and the autotransporter pertactin (Prn) have been included in acellular vaccines, yet PT remains central in disease and immunity. 

Comparative genomics may provide insights into the role of PT in disease. *B. pertussis* is closely related to *B***.** *parapertussis* and *B. bronchiseptica*, with *B***.** *parapertussis* causing a similar, but milder version of disease in humans and ovines [27], and *B. bronchiseptica* infecting many mammals, including dogs and swine, but very rarely infecting humans [28]. Only *B. pertussis* is known to express PT. Both *B. parapertussis* and *B. bronchiseptica* have homologous regions encoding functional PT, which can be expressed when engineered with a *B. pertussis* promoter region [29,30]. The driving forces favoring the loss of PT expression by *B. parapertussis* and *B. bronchiseptica* are unknown. These genetic, host, and disease similarities between *B. pertussis* and *B. parapertussis* suggest that PT exacerbates disease, but is not necessary for symptoms, including coughing. Unfortunately, these direct comparisons are tenuous since *B. parapertussis* evolved to infect humans independently from *B. pertussis* [31], so their mechanisms of causing human disease may be different. Another example of a bacterium that has very different mechanisms of infection from another genetically similar bacterium is *Yersinia pestis*. *Y. pestis* is taxologically similar enough to be a subspecies of *Y. pseudotuberculosis* [32,33,34], yet *Y. pestis* is vector- or aerosol-borne and causes primarily bubonic, septicemic and/or pneumonic disease, whereas *Y. pseudotuberculosis* is food-borne and causes a primarily enteric disease [35]. This illustrates that similar disease mechanisms cannot be assumed in closely related species like *B. pertussis* and *B. parapertussis*. Within *B. pertussis*, only two pertussis-toxin deficient *B. pertussis* strains have been isolated from patients. One was isolated in 2007 from a 3-month-old infant in France, and the second was isolated in 2013 from an 11-month-old infant in New York, USA. Both infants had classic symptoms of pertussis and both contained the same deletion of the PT locus between two insertion elements [36,37]. Again, this data suggests, but does not prove, that PT is dispensable for disease. These retrospective anecdotes do not eliminate the possibility of co-infections with a PT-positive pertussis strain or the possibility that these individuals were infected with a wild-type strain and the deletion mutant arose during the course of infection. Therefore, these examples do not reliably inform us on the role of PT in infection. Clearly, PT-deficient clinical isolates are extremely rare. Given the large size of the locus encoding PT and the toxin-secretion apparatus, PT-deficient clinical isolates would be expected to occur at a relatively high rate if PT was dispensable for infection. 

PT affects many cell types and it has been shown to have many, and sometimes seemingly contradictory effects in vivo and in vitro. This includes functioning as both a pro-inflammatory adjuvant [38,39] and anti-inflammatory agent [39,40], inhibiting macrophage and neutrophil migration [40,41] and phagocytosis [42], and reducing or potentiating vascular permeability in response to different molecules [40]. These studies may not reflect the true effects of PT expressed during a natural pertussis infection, given differences in concentrations as well as anatomical and temporal exposure. Direct comparisons between wild-type and PT-knock out pertussis infections in mice have provided more relevant insight into PT’s role in pathogenesis. In this model, PT hinders early innate immune responses in part by targeting alveolar macrophages to promote *B. pertussis* infection [17] and delay neutrophil recruitment to the lung [18]. It does this by inhibiting cytokine and chemokine production by aveolar macrophages and other lung cells [43], not by direct action on neutrophils, as was previously thought [40]. PT has also been shown to play a role in resistance to antibody-mediated clearance of *B. pertussis* [18], and in suppression of antibody production against *B. pertussis* antigens, including FHA [44,45]. Later in infection, PT promotes and prolongs the inflammatory response in the lung [46,47]. In neonatal mice, PT reduced lung pathology and increased mortality. The authors suggest that infant mortality from *B. pertussis* may be due to systemic effects of PT, and not from lung pathology [14]. 

In a rat model of whooping cough, wild-type *B. pertussis* strains induced coughing but a strain lacking PT did not, providing direct evidence that PT is required for coughing in that model [48]. Additional studies in a more relevant model, such as in humans or the baboon model of pertussis are needed to directly address the role of PT in pertussis-induced coughing. No coughing was observed in a clinical study in which purified, fully-active PT was injected into subjects at a dose of 1 µg/kg to evaluate PT’s potential use as a therapeutic to increase insulin sensitivity in diabetics [49]. This suggests that PT alone does not induce coughing, however, the immunization status of these individuals was not reported and it is not known how the injected, bolus dose of PT compares to an exposure resulting from infection. An intravenous PT dose of 12.5 µg/kg did not induce leukocytosis in rhesus macaques [50], whereas a 25 µg/kg caused leukocytosis to levels similar to those observed in clinical disease [1,2,51]. Higher intravenous doses of PT alone is toxic in mice and rats, causing weight loss, “a puffy face” (suggestive of enhanced vascular leakage and edema), splenic and thymic atrophy, decreased activity, tearing, and death with a respective mean lethal dose of 127 µg/kg and 114 µg/kg [49].

## 3. PT as Protective Antigen 

Investigation into the protective immune response against pertussis began shortly after *B. pertussis* was identified as the causative agent of pertussis. Polyclonal anti-toxin immune therapies were investigated, with immune serum generated against the whole pathogen or against multiple soluble factors) [6]. Uncontrolled case studies of these non-standard therapies had mixed results [52]. To standardize the treatments, researchers isolated protein fractions from *B. pertussis* to try and find the agglutinizing toxin, a protective antigen identified in the 1940s [53]. A protein fraction with hemagglutinizing and protective properties was found in some, but not all studies [54,55,56,57], which could have been due to differences in growth and expression of PT and method of pertussis challenge. This protein fraction was actually composed of two hemagglutinating proteins—fimbrial hemagglutinin, now known as filamentous hemagglutinin (FHA) and leukocytosis-promoting factor hemagglutinin, now known as PT. Different research groups isolated PT from *B. pertussis* culture supernatant, free from FHA contamination, in order to help elucidate which fraction, or if both fractions, were protective [58]. Pure PT was toxoided with formalin and was successfully used to immunize mice against a lethal intracerebral (IC) challenge of *B. pertussis*—pure FHA was not protective in the IC model [20,59]. The IC challenge model was a commonly used assay at the time since it was predictive of pertussis-vaccine efficacy in field trials [60]. Passive immunization with PT-antisera also protected mice from IC challenge [20,59], and monoclonal antibodies raised against pure PT were protective against disease in mice by both IC and aerosol challenge [61]. Sato et al. found that the PT fraction had the most protective effects in mouse models of pertussis and FHA was able to boost the immunogenicity of PT [62]. Results from monoclonal antibody prophylaxis of mice with anti-PT or anti-FHA antibodies prior to aerosol challenge were similar, with anti-FHA prophylaxis being protective, but not quite as effective as anti-PT pretreatment in reducing symptoms [63]. These data supported the inclusion of both FHA and PT in the Japanese acellular vaccine [62,63]. Both PT and FHA are protective in a murine aerosol challenge model of pertussis [20,59,63]. Additional studies found that FHA alone [64,65], or an FHA epitope [66], conferred protection when delivered mucosally. While mouse studies were instrumental in helping develop the acellular pertussis vaccines, it is important to not draw too many conclusions about the protective efficacy of a given antigen based on mouse models alone. *B. pertussis* is a human-specific pathogen and mice do not fully capitulate the human course of disease. Additionally, FHA may play a role in host specificity [67], indicating that murine studies on the protective nature of FHA are additionally confounded and protective antigens identified in the mouse model may not reflect the true human protective antigens. Human studies, or studies conducted in the closely related baboon model, may provide more relevant data regarding the protective effects of individual antigens.

Clinical studies investigating pertussis vaccine serologic correlates of protection identified antibodies against PT, Fim2/3 and pertactin as protective in two efficacy trials with the aP vaccine but antibodies against FHA were not [68,69]. In contrast, a small, prospective study conducted during a pertussis outbreak in Finnish children who were immunized with the Finnish wP DTP vaccine found that children with high levels of anti-FHA antibody were protected [70]. 

Protection from clinical disease does not correlate with protection from colonization or transmission. An epidemiological study in England and Wales found that the frequency of pertussis outbreaks did not decrease following the widespread use of wP vaccines, nor did it increase during a fall in the vaccination rate as would be expected. This stable epidemic frequency is suggestive of a vaccine that prevents clinical disease, but does not prevent colonization and transmission [71]. Later epidemiological analysis with a more extensive data set did observe a decrease in epidemic frequency in England and Wales [72], and a pertussis vaccine efficacy study in Senegal showed reduced transmission of disease in primarily wP vaccinated children during a pertussis epidemic [73]. A vaccination study in infant baboons appears to support the 1982 epidemiological data. Infant baboons vaccinated with either a wP or an aP vaccine (containing PT, FHA, Prn and Fim2 and 3) were protected from disease when directly challenged with *B. pertussis*. WP and aP-vaccinated animals were colonized upon challenge, but the wP group cleared the infection more quickly than the naïve, un-vaccinated animals, and the aP-vaccinated animals remained colonized for longer than the naïve, un-vaccinated animals. Additionally, the aP-vaccinated animals were colonized to the same level as naïve animals following natural transmission and transmitted disease to unvaccinated animals [74]. It is possible that aP vaccination reduces transmission by reducing rhinorrhea and coughing but these baboon studies indicate that aP vaccination does not prevent colonization or transmission. 

## 4. Pertussis Toxin-Only Vaccination and Inactivation

Prevention of disease with the use of PT-only vaccines has been demonstrated in humans. A 1988 placebo-controlled study found that a mono-component PT vaccine was estimated to be 54% effective, while a two-component PT and FHA vaccine was slightly more effective at 69%, similar to the Sato et al. studies discussed earlier [62,63]. Both of these vaccines were 80% effective in preventing more serious disease defined as culture-confirmed pertussis lasting more than 30 days [75]. Another mono-component PT vaccine study was completed in Gothenburg, Sweden in a previously unvaccinated population. Vaccination with the PT-only vaccine decreased the incidence of pertussis in vaccinated children with an efficacy of 71%, similar to the PT and FHA-containing vaccine. It also decreased pertussis cases in vaccinees’ household contacts and in older, unvaccinated children, suggesting that use of the PT-only vaccine induced a level of herd immunity. Importantly, the study found that protected vaccinees had higher levels of PT-specific antibodies, whereas high levels of FHA- and pertactin-specific antibodies may have been elicited by other Bordetellae infections [76]. The researchers in the Gothenburg study concluded that PT “is both an essential and alone sufficient antigen in acellular pertussis vaccines [76].” However, after three years of surveillance, protection in the PT-only group was lower than that observed in the two-component vaccine group [77]. An analysis of multiple vaccine efficacy trials by Storsaeter et al. found that levels of anti-PT, pertactin and Fim 2/3 antibodies, but not FHA were associated with protection, but the authors urge readers that their analysis “should not be overinterpreted and should not be taken to indicate a proven causal relationship [68],” especially in light of an Italian vaccine efficacy study. This study observed good levels of protection from aP vaccination despite quickly waning antibody responses against PT, FHA and pertactin, suggesting that immunity not measured by a serologic response plays a role in protection [78,79]. A PT-only vaccine has been in use for over 15 years in Denmark and it has been very effective, further supporting the efficacy of a PT-only vaccine. Evidence from the baboon model of pertussis suggests that PT-only vaccines are also effective trans-placentally. Five-week old infant baboons directly challenged with *B. pertussis* were protected from disease if their mother had been vaccinated with a PT-only vaccine during pregnancy, but these infants were still colonized. It is reasonable to conclude that these infants were protected by maternal antibodies transferred during pregnancy, supporting the conclusion that antitoxin alone is sufficient to protect against disease, but not colonization [80]. 

The method of PT inactivation and stabilization is important for vaccinology since altering the molecular structure of PT, via chemical modifications, heat or genetically, can alter its antigenicity. This further confounds comparisons between pertussis vaccines with different formulations. These alterations may affect conformational epitopes, the 3-dimensional surface(s) recognized by B-cell immunoglobulins, and the processing and presentation of linear epitopes, the peptides presented by antigen presenting cells to helper CD4 and killer CD8 T cells. Many chemical detoxification methods have been used, including formaldehyde, glutaraldehyde, hydrogen peroxide, and tetranitromethane [76,81]. All chemical methods of detoxification have the potential to decrease antigenicity and allow for reversion to a toxic molecule. Researchers suggest that the efficacy of the Denmark mono-component vaccine may lie in its formulation. It includes a higher dose PT that is inactivated with hydrogen peroxide, a different method of inactivation than the more commonly used formaldehyde. Studies indicate that hydrogen peroxide inactivation of PT preserves more critical epitopes for antibody recognition than formaldehyde [82,83]. Formaldehyde treatment to inactivate PT is known to alter both conformational and linear epitopes, which could reduce vaccine effectiveness [84,85]. Sutherland et al. demonstrated that the antibody responses to formaldehyde detoxified PT in a vaccine differed to those produced by natural infections, with the natural infection producing more antibodies against protective conformational epitopes, suggesting that alternate methods of detoxification could allow for more effective vaccines [85]. To address safety concerns and enhance immunogenicity, Locht et al. [86,87,88] and Pizza et al. [89] have introduced genetic mutations to alter key residues of the S1, or A subunit of PT, resulting in genetically inactivated pertussis toxin. Studies by Pizza et al. show that their genetically inactivated PT is only immunogenic when produced as a holotoxin. This is potentially due to the disordered nature of the A subunit and the stabilization of conformational epitopes by the B_5_ oligomer. The genetically inactivated PT maintained both T- and B-cell epitopes [89]. A genetically detoxified PT made by Biocine (Siena, Italy) induced high-titers of anti-PT antibodies in a clinical trial comparing serologic responses between 13 different aP vaccines, despite using roughly one half to one-fifth the amount of PT as most of the other aP vaccines [81]. In a 2015 phase 2 and 3 non-inferiority vaccine trial, vaccines containing either genetically inactivated PT and FHA only, or genetically inactivated PT, FHA, D and T, was shown to induce significantly more seroconversion (approximately 40% for PT and 30%–40% for FHA) and significantly higher geometric mean titers and anti-PT neutralizing antibody titers when compared to a TdaP vaccine. This study led to the licensure of Pertagen (aP_(PTgen/FHA)_) and Boostagen (TdaP_(PTgen/FHA)_) in Thailand [11]. The availability of a more effective pertussis-only aP vaccine, such as the Thai Pertagen vaccine, as opposed to TdaP combination vaccines, could increase maternal vaccination rates [10,90].

Further characterization of T- and B-cell epitopes in PT, both from natural *B. pertussis* infections and vaccination, is needed to help better select PT-inactivation and stabilization methods, preserve potential protective vaccine epitopes, and better understand waning protective immunity to aP vaccination [91,92]. It is also important to consider adjuvants in vaccine formulations. Adjuvants can play an important role in directing the vaccine-induced immune response to pertussis, and may improve immunogenicity in next-generation aP vaccines [93,94].

## 5. Anti-Pertussis Toxin Immunoglobulin Therapies in Humans and Non-Human Primates

Assuming a serologic response is sufficient to neutralize PT and protect against disease, immunoglobulin therapies targeting pertussis should be protective and clearly identify PT titers as protective. Unfortunately, studies investigating this question are not conclusive. A placebo-controlled trial was conducted in the 1970s on an anti-PT immunoglobulin administered intramuscularly to patients within the first week of paroxysmal coughing. The treatment was not effective at reducing symptomology, but this may have been due to the intramuscular administration, which does not result in rapid attainment of peak antibody concentrations in the blood [95]. Addressing this concern, another study utilized intravenous administration of a high-titer polyclonal human anti-PT IgG (P-IVIG), prepared from sera harvested from individuals vaccinated with a pertussis toxoid. This study saw non-statistical decreases in paroxyms and white blood cell counts following P-IVIG treatment of children with pertussis, when compared to pretreatment values [96]. However, this study was a small phase I clinical trial designed to examine dosing and safety, not efficacy. A phase 3, randomized, placebo-controlled clinical trial of P-IVIG was discontinued due to a slow rate of enrollment [97]. To address the concerns of availability and variability of human polyclonal anti-sera, Nguyen et al. [98] humanized two well-characterized PT-neutralizing mouse monoclonal antibodies developed by Sato et al. [61]. These monoclonal antibodies target epitopes on the S1 or the S2 and S3 subunits of PT and are believed to respectively inhibit the ADP-ribosylating and target-cell binding capabilities of PT [61,99]. These antibodies are thought to bind epitopes similar to antibodies elicited during a natural pertussis infection, and are therefore more protective than antibodies raised against a chemically toxoided PT [85], the antigen used to generate P-IVIG. These humanized antibodies were used to treat weanling baboons infected with pertussis. Unfortunately, two of the four antibody-treated animals had previous exposure to *Bordetella bronchiseptica*, as evidenced by their anamnestic antibody response to FHA, and were partially protected from the *B. pertussis* infection [100]. The two remaining animals in the monoclonal antibody-cocktail treatment group appeared to have decreased coughing and leukocytosis as compared to the control animals, suggesting that passively administered antitoxin is sufficient to confer protection against symptoms [98]. This further supports the baboon maternal PT-only vaccination data, in which antibody transfer against PT is sufficient to protect infants from infection [80]. 

Additional research is needed to determine the efficacy of immunoglobulin therapies against PT, and if antibodies against different *B. pertussis* antigens could be used alone or in combination with anti-PT antibodies to boost its therapeutic potential. It is also possible that a more cellular-skewed or a mucosal-type (IgA) antibody response would more effectively prevent disease or clear infection, highlighting the need for a better understanding of the protective immune response to a *B. pertussis* infection. 

## 6. Discussion

Clearly, PT is a key protective antigen in pertussis vaccines, despite the lack of a full understanding of its mechanism of action. PT is responsible for specific pertussis symptoms, but by itself does not recapitulate the full spectrum of disease. This is evidenced by the effectiveness of PT mono-component pertussis vaccines in preventing disease [76,77,80,101] and the lack of overt pertussis symptoms upon injection with PT [49]. Challenging adult humans [102] or infant baboons with a PT knock-out strain of *B. pertussis* is required to directly address the role PT plays in the establishment of disease and/or disease progression, given the host specificity of *B. pertussis*. Human challenge studies could address the role of PT in colonization and early symptoms. Baboon studies could directly assess the role PT has in many aspects of disease pathogenesis, including pertussis-induced coughing, dissemination and colonization of the lungs and upper respiratory tract, systemic dissemination (if any), lung inflammation and damage, pulmonary hypertension, skewing of the host immune response(s), and other systemic perturbations. It is currently difficult to draw cross-study conclusions regarding the relative efficacy of different antigens present in pertussis vaccines given the many differences in manufacturing and formulation. The human and baboon challenge models may also be able to determine the protective effects that additional vaccine antigens, adjuvants, formulations or delivery routes may have on disease symptomology and on colonization and transmission. Transcriptomic, proteomic, metabolomic and other -omics studies in these models may also help identify pertussis correlates of protection, facilitating vaccine development and manufacture. 

Despite our imprecise understanding of PT after over a century of investigation, PT is clearly a critical virulence factor for *B. pertussis* and immune responses to PT protect against disease. PT will likely remain a key component in the next generation of pertussis vaccines.
